# First report on establishment and characterization of a carcinosarcoma tumour cell line model of the bladder

**DOI:** 10.1038/s41598-021-85400-5

**Published:** 2021-03-16

**Authors:** Johannes Eberhard, Daniela Hirsch, Oliver Schilling, Wilhelm G. Dirks, Feng Guo, Alice Fabarius, Felix Rückert, Christoph Reißfelder, Peter Hohenberger, Prama Pallavi

**Affiliations:** 1 Surgical Department, University Hospital Mannheim, Heidelberg University, Theodor-Kutzer-Ufer 1-3, 68167 Mannheim, Germany; 2 Institute of Pathology, University Hospital Mannheim, Heidelberg University, Mannheim, Germany; 3 Institute of Surgical Pathology, University Medical Center Freiburg, Faculty of Medicine, University of Freiburg, Freiburg, Germany; 4 Department of Human and Animal Cell Lines, Leibniz-Institute DSMZ-German Collection of Microorganisms and Cell Cultures, Braunschweig, Germany; 5 Department of Haematology and Oncology, University Hospital Mannheim, Heidelberg University, Mannheim, Germany; 6 Division of Surgical Oncology and Thoracic Surgery, Medical Faculty Mannheim, University Hospital Mannheim, University of Heidelberg, Mannheim, Germany; 7 Deutsches Konsortium für Translationale Krebsforschung, Standort Freiburg, Freiburg, Germany

**Keywords:** Cancer models, Urological cancer, Oncology, Surgical oncology

## Abstract

Carcinosarcoma of the urinary bladder is a very rare and aggressive subtype of bladder cancer with poor prognosis. Characteristically carcinosarcomas exhibit biphasic nature with both epithelial and mesenchymal differentiation. Limited information is available regarding its clinical features and appropriate treatments due to its rarity. Development of tumour models can further our understanding of bladder carcinosarcoma. We report establishment and characterization of the first-ever bladder carcinosarcoma cell line MaS-3. It is established by the outgrow method from 86 year-old caucasian male who underwent a radical pelvic resection after neoadjuvant radiotherapy. MaS-3 showed carcinosarcoma profile with high conformity with to the original tumour in terms of immunocytochemistry. Proteome analysis also aligned the MaS-3 cell line with the carcinosarcoma specimen rather than corresponding non-malignant tissue. Chemotherapy sensitivity testing revealed a great sensitivity of MaS-3 growth to 5-Fluorouracil, Gemcitabine and Cisplatin, with almost no impact of Irinotecan. Additionally, the suitability of MaS-3 for 3D in vitro experiments was also demonstrated. The newly established cell line MaS-3 shows typical characteristics of the tumour and may thus be a useful in vitro model system for studying the tumour biology and developing future of treatments of this rare but very aggressive entity.

## Introduction

Carcinosarcomas are an uncommon malignancy which is characterized as a dedifferentiated biphasic tumour that exhibits morphological and immunohistochemical evidence of both epithelial and mesenchymal differentiation in distinction of carcinomas or sarcomas^1^. Other terms for this tumour entity are sarcomatoid carcinoma, spindle cell carcinoma or metaplastic carcinoma^2^. Carcinosarcomas might arise in in different organs like the oropharynx, the lung, the male genital system and the female reproductive organs with the so-called Malignant Mixed Müllerian tumour, the most often detected type of carcinosarcoma^3,4^. Carcinosarcomas within the bladder are very rare, accounting for only approximately 0.11% of all primary bladder tumours^5^. Epidemiologically carcinosarcomas of the bladder are a tumour entity that occurs more often in elder males in their seventh decade^6,7^. Risk factors for carcinosarcoma a typically are those for urothelial carcinoma, such as recurrent cystitis, diabetes and especially smoking^6–8^.

Due to its rarity the understanding of this disease entity is limited. A systematic literature search for published reports resulted in the identification of 276 reports published from January 1960 to January 2014. No prospective study or clinical trial was reported in English language^9^.

Carcinosarcomas have a dismal prognosis, more than 70% of cases present with advanced stage and the 5 year survival rate is 17 to 20% after resection only^6,7,10^. Radical surgery, which means total cystectomy in most cases, followed by radiation or chemotherapy is recommended, although evidence to support this regimen is very low due to limited case numbers^11,12^.

The distinction and origin of bladder carcinosarcomas is still subject of an ongoing debate. (Histo-)Morphologically the differential diagnosis between carcinosarcoma and other tumour entities of the bladder may be very challenging as the two elements may show gradual coalescing or the sarcomatous component has outgrown the epithelial component^13^. The mixed epithelial and mesenchymal cell elements immunohistochemically express keratins and epithelial membrane antigen (EMA) and mesenchymal markers such as vimentin^10,14^. Some authors reported that the sarcomatoid components of carcinosarcomas completely lack epithelial cell surface markers and ultrastructural features^4^. However, Ikegami et al. showed that both carcinomatous and sarcomatoid components expressed remnant markers of epithelial differentiation to a varying degree, namely pankeratin and epithelial membrane antigen^14^. While vimentin is expressed in the sarcomatous component in almost all cases, the expression of epithelial markers may vary, making it difficult to distinguish from true sarcomas^4,13,14^. The immunohistochemical evidence of cytokeratin AE1/AE3 seems to be the most sensitive marker^15^. There are different theories how such biphasic tumours develop. Previous studies have shown that the mesenchymal and epithelial components might be of monoclonal origin^16,17^. Hence, the theory that carcinosarcomas are a “collision-tumour” of two independently and synchronously developed mesenchymal and epithelial tumours of multiclonal origin that “collide” in the same organ, has been more and more abandoned^13^. The monoclonal origin however is not yet fully understood. It was suggested that the tumour could either be due to a multi- or pluripotent tumour stem cell which descendant cells might differentiate into both, epithelial and spindle cell components^18^. In contrast, another more widely accepted theory assumes that an epithelial cancer dedifferentiates maybe under the influence to the stromal microenvironment into a mesenchymal phenotype^13^.

Due to its rarity and not yet fully elucidated carcinogenesis uncertainty still exists regarding the clinical and therapeutic implications of this entity. For an in depth understanding basic research is essential. Therefore, the aim of the present study was to establish the world’s first tumour model of this entity from a histologically proven carcinosarcoma of the bladder and to characterize its use for research comprehensively.

## Results

### Morphology and growth characteristics

**Figure 1:**
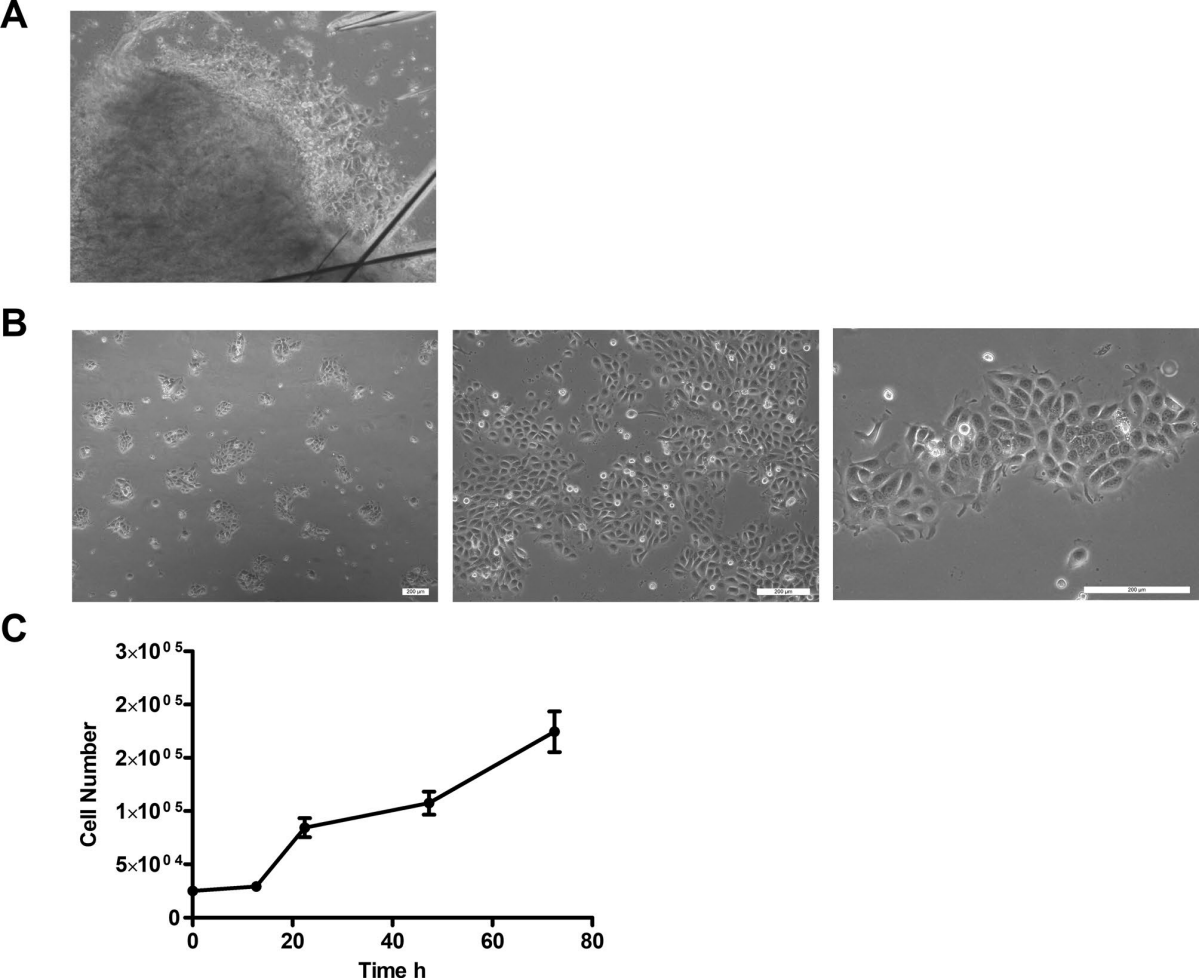
MaS-3 shows a distinct morphology. (**A**) Cells growing out of tumour fragment 4 days after seeding. (**B**) Morphology of MaS-3 cells passage 10 under phase contrast microscope (a) ×5, (b) ×10, (c) ×20. (**C**) Growth curve of MaS-3 cells. Error bars indicate standard error of mean.

MaS-3 grows in a typical monolayer in a cobblestone-like and insular pattern with well demarked cell boundaries. On the second day of the explant culture tumour cells outgrew and formed small colonies (Fig. 1A). The morphological appearance is epithelial-like cells arranged in the form of a jigsaw puzzle mixed with mesenchymal-like cells with a spindle-shaped or fibroblast-like morphology (Fig. 1B)*.* The doubling time was estimated to be 26 h (Fig. 1C).

A search of the STR profile within the international STR reference database performed by DSMZ revealed uniqueness of MaS-3. Furthermore, the concordance of the identity of the cell line and the primary tumour it was derived from was confirmed by an ID panel (CODIS/ESS) (Supplementary Figure 1).

### Immunocytochemistry

**Figure 2:**
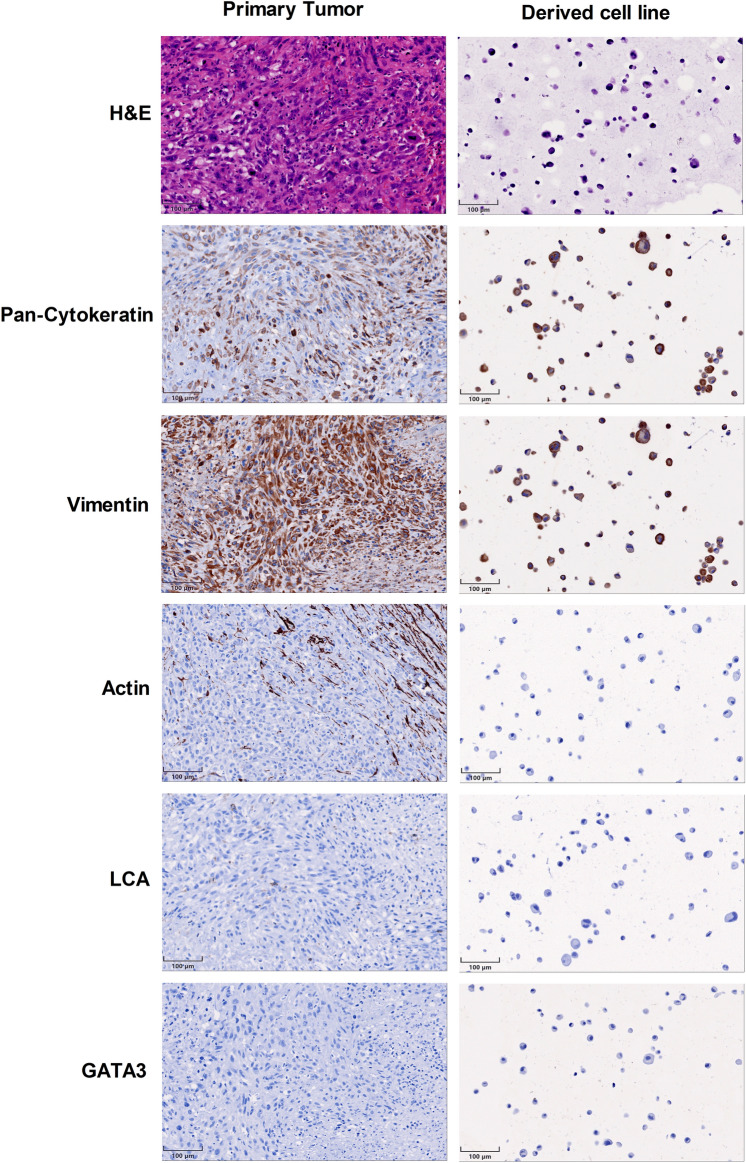
MaS-3 shows similar expression pattern as original tumour tissue Immunohistochemistry of MaS-3 cell line and original tumour.

The newly established cell line closely resembled the immunohistochemical staining profile of the tumour from which it has been derived (Fig. 2). In line with the primary tumour, cultured cells maintained expression of pancytokeratin as well as vimentin while actin was consistently not expressed. Leukocyte phenotype was excluded by LCA/CD45 staining. Of note, in contrast to the pre-treatment biopsy, the primary tumour lost GATA-3 expression after neoadjuvant therapy and consistently, the cell line, which was established from the post-treatment resection specimen, did not show GATA-3 expression either. In line, STR assay also proofed the cell line to be derived from the native tumour (Supplementary Figure 2).

### Cytogenetics and multicolour fluorescence in situ hybridization analysis

Cytogenetic analysis of MaS-3 revealed a hypotriploid to hypertriploid male karyotype with several numerical and structural chromosomal alterations like gains of whole chromosomes and structural changes like translocations or deletions. The composite karyotype (cp) contains all clonally observed abnormalities and gives a range of chromosome number in the metaphases. The total number of cells in which the clonal changes were observed is given in square brackets after the karyotype, preceded by the symbol cp. Each of the abnormalities has been analysed in at least two metaphases, but there is no cell with all abnormalities. Sporadic chromosomal aberrations (observed only in one metaphase) are not mentioned according to the ISCN 2016.

$$\begin{aligned} & 56\sim75,{\text{XXY}},{\text{t}}\left( {{\text{Y}};21} \right), + 1,{\text{t}}\left( {1;4} \right),{\text{t}}\left( {1;10} \right), \\ & \quad + 2,{\text{del}}\left( {2{\text{q}}} \right),{\text{t}}\left( {2;16} \right), + 3, + {\text{t}}\left( {3;14} \right), + {\text{t}}\left( {3;15} \right), \\ & \quad + {\text{t}}\left( {3;18} \right), + 5, + 5,{\text{del}}\left( {5{\text{q}}} \right), + 6, + 7, + 7, + {\text{i}}\left( 8 \right), + {\text{i}}\left( 8 \right), \\ & \quad + {\text{t}}\left( {8;17} \right), + {\text{t}}\left( {8;17} \right),{\text{del}}\left( {9{\text{q}}} \right), + 10, + 10, + 11,{\text{t}}\left( {11;1;11} \right), \\ & \quad + {\text{dup}}\left( {11{\text{q}}} \right), + {\text{dup}}\left( {11{\text{q}}} \right), + 12, + 13, + 13,{\text{t}}\left( {13;22} \right), \\ & \quad + {\text{t}}\left( {14;18} \right), + 16, + 18, + 19, + 19, + 20[{\text{cp}}20] \\ \end{aligned}$$

### Chemosensitivity

**Figure 3:**
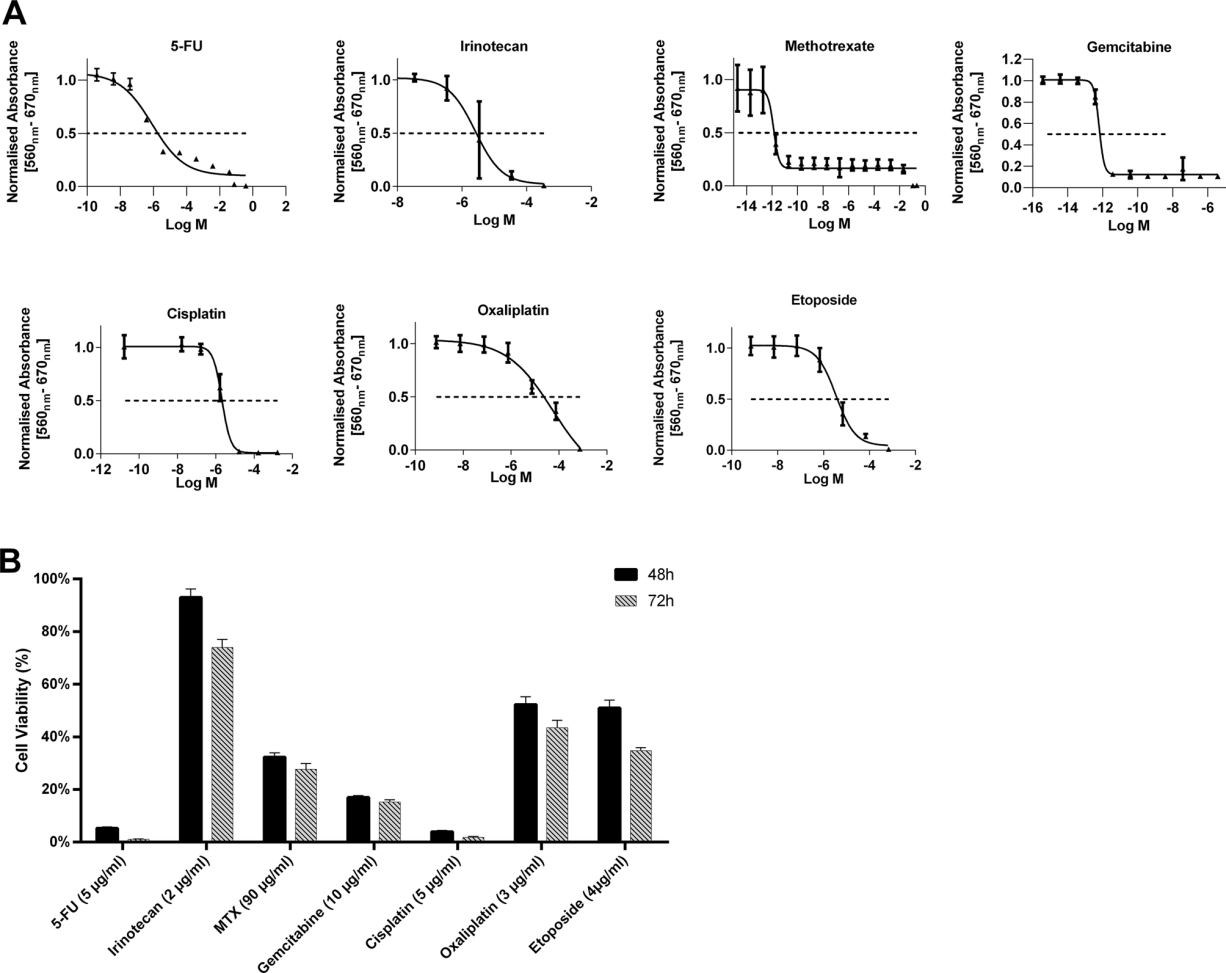
MaS-3 is most sensitive to Methotrexate, Gemcitabine and Cisplatin. (**A**) IC_50_ dose response curves of MAS-3 to 48 h treatment 5-Flourouracil, Irinotecan, Methotrexate Gemcitabine, Cisplatin, Oxaliplatin and Etoposide as calculated by non-linear regression in GraphPad Prism ver 8.0. (**B**) Chemosensitivity of MaS-3 cell line to the mean serum value of tested chemo drugs as identified by pubchem search (https://pubchem.ncbi.nlm.nih.gov) after 48 and 72 h of treatment. All sets were normalized to untreated controls (mean value = 1). Error bars represent standard deviation. All experiments were performed with atleast three different passages of MAS-3 and within each experiment there were six technical replicates.

A broad variety of different agents that are routinely used for cancer treatment were tested in a dose–response manner. The tested range was based on their usual plasma levels according to pubchem search (https://pubchem.ncbi.nlm.nih.gov). All tested agents led to a reduction in MaS-3 growth. The IC_50_ values are shown with the response curves (Fig. 3A). Effectivness of the tested drugs is as follows Gemcitabine (IC_50_: 0.64 pM), Methotrexate (IC_50_: 1.35 pM), 5-FU (IC_50_: 0.84 µM), Cisplatin (IC_50_: 2.11 µM), Irinotecan (IC_50_: 2.57 µM), Etopside (IC_50_: 3.45 µM µg/ml) and lastly Oxaliplatin (IC_50_: 50 µM). Although IC_50_ values for Gemcitabine and Methotrexate were lowest, yet a certain subset of cells (nearly 20%) showed resistance and survived at very high concentration. All drugs except Irinotecan showed a significant reduction in cell proliferation compared to control at usual serum concentrations after 48 h (Fig. 3B). The treatment with 5-FU and Cisplatin even caused an almost complete death of all cells seeded. 72 h treatment yielded an additional decrease in cell proliferation compared to control and was observable for all tested reagents except Gemcitabine.

### Proteomics

**Figure 4:**
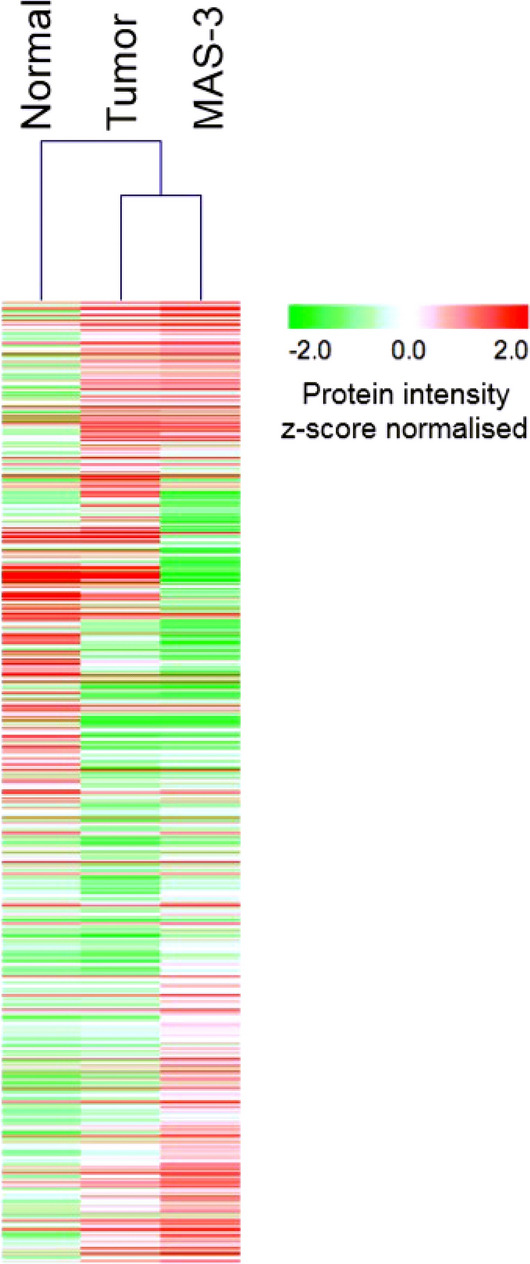
Proteomics analysis of MaS-3 cells revels carcinosarcoma nature. Hierarchical clustering of the core proteome of MaS-3 cells in comparison to carcinosarcoma and corresponding non-malignant tissue.

Mass spectrometry based proteomics of MaS-3 cells in comparison with a carcinosarcoma specimen and corresponding non-malignant tissue enabled the identification (false-discovery rate < 1%) and quantification of more than 600 proteins in all three samples. For improved comparability, this analysis omitted tissue-specific proteins, such as blood-specific proteins, which are typically absent in cultivated cell lines. With the core set of 600 proteins we performed hierarchical clustering (HCL, Fig. 4). In HCL analysis, the MaS-3 tumour cell line aligned more closely with the carcinosarcoma sample than with the corresponding non-malignant tissue sample. We consider this finding to further support suitability of the MaS-3 cell line model for in vitro studies of carcinosarcoma. We further employed protein set enrichment analysis to classify altered protein abundances of carcinosarcoma sample and the corresponding non-malignant tissue sample. We notice an increase of cytoskeletal proteins in the tumor sample with a corresponding decrease of extracellular matrix components (not shown). Protein expression studies of bladder carcinosarcoma have remained scarce. Two studies emphasize noticeable expression of vimentin, which is reflected by the MaS-3 cell line^19,20^. Ikegami et al. report expression of CD44 in bladder carcinosarcoma, which we also observe in the proteomic analysis of the MaS-3 cell line^14^. Likewise, MaS-3 cells express HPS70 and protein S100, whose expression Kamishima et al. have equally determined for bladder carcinosarcoma^20^.

### 3D cell culture

**Figure 5:**
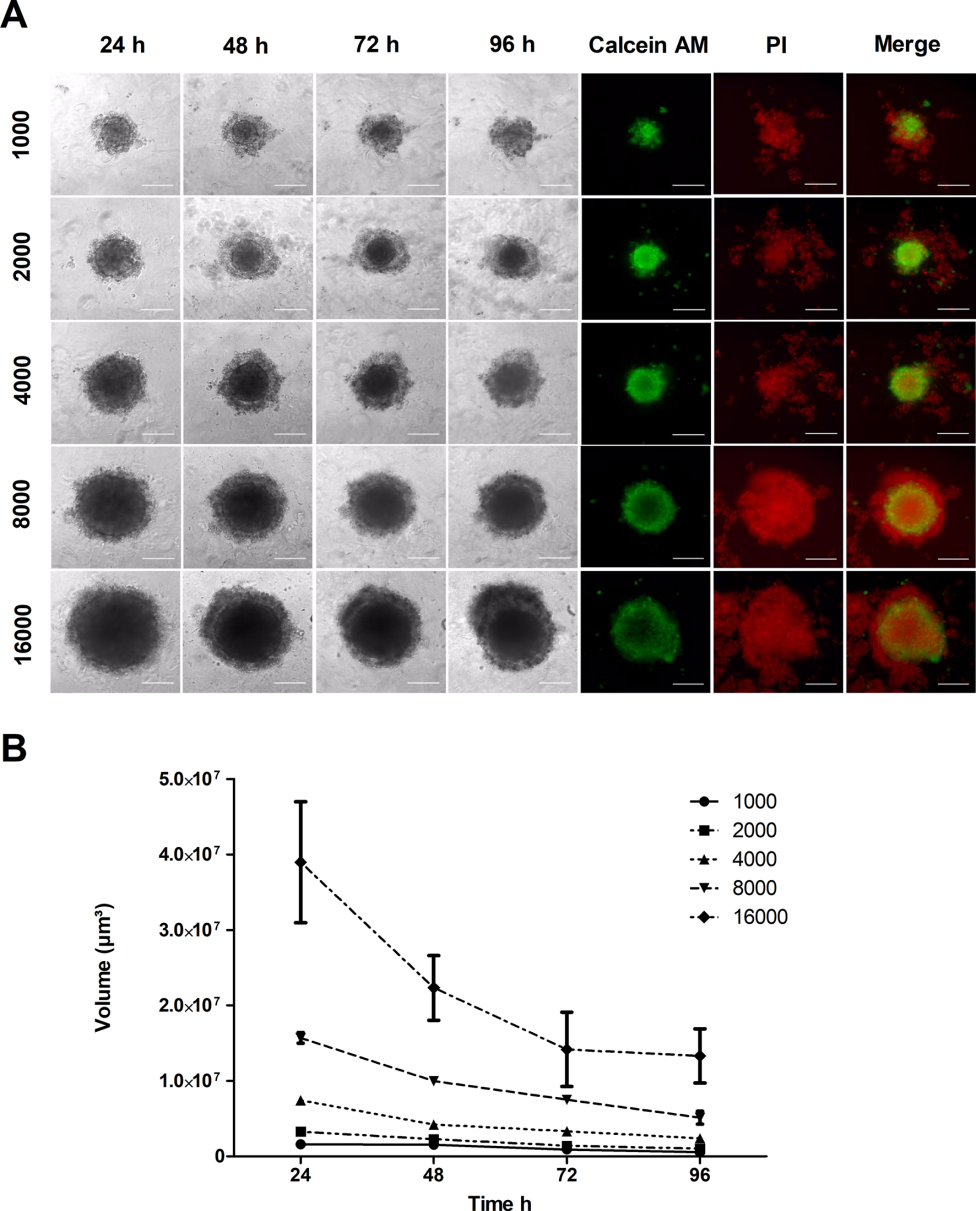
MaS-3 cell line can grow in 3D as spheroids. (**A**) Spheroids were obtained with seeding different cell densities of MaS-3. Post 96 h live/dead staining with calcein AM (green), ethidium homodimer-1 (red) and merged was performed. Flurosent images were taken with ×10 magnification on a DM IRB Leica microscope. Scale bars denote 200 µm. (**B**) Volume curve for MaS-3 spheroids over time. Experiment was performed in triplicates error bars denote standard error of mean.

24 h after seeding MaS-3 cells formed spheroids in all seeding densities with a distinct border and a diameter up to 500 µm (16,000 cell seeding). Usage of matrigel did not improved spheroid formation (Supplementary Figure 4). Over time the spheroids became more compact (Fig. 5A) and decreased in volume. The volume reduction as specifically observable in the higher seeding concentrations (Fig. 5B). Calcein AM and Propidium iodide staining revealed that after 96 h cells were still growing in a viable structure, surrounded by some dead cells. In higher seeding concentrations the core also showed a decreased cell viability (Fig. 5A).

## Discussion

According to RARECARENet, rare tumours are defined as less than 6 newly diagnosed cases per 100,000 inhabitants. Rare tumours are correlated with an unfavourable prognosis mainly because they are more often misdiagnosed as physicians may only see a very rare cancer once or twice in their career^21^. Also, successful clinical and experimental research is hindered by the limited availability of the biological material. The latter is of importance for experimental research because the understanding of the tumour physiology might lead to the development of better treatment options. Cell lines are a nearly unlimited source for tumour-derived proteins, DNA and RNA, and might be used for in vitro modelling of cancer subtypes. In the present article we describe the isolation and characterization of MaS-3, which to our knowledge, is the only cell line originating from bladder carcinosarcoma.

Cell lines are a prerequisite for basic research on tumour pathology and treatment options, as they are an economic and easily operated tumour model. To investigate the characteristics and proof the origin and biphasic nature of the cell line we performed various analyses. Microscopically, we found that the morphology of MaS-3 resembles its suggested biphasic nature. Cells had a rather lose cell–cell contact and grew in colonies. The relatively high doubling rate resembles the high-grade malignancy of the tumour.

Immunocytochemical characteristics were highly concordant between the primary tumour and the newly established cell line. However, after neoadjuvant treatment the tumour lost expression of GATA-3. The expression of GATA-3 in the tumour biopsy before neoadjuvant treatment indicated urothelial origin of the carcinosarcoma.

To further investigate the clinical characteristics of the new cell line we tested its response to a wide range of chemotherapeutics. The effect of radiotherapy was not tested, as the patient underwent neoadjuvant radiotherapy and tumour cells sensitive to radiation might have perished before the specimen from the tumour host was taken. However because of the possible impact of the in vivo microenvironment, the cell line still could be sensitive to radiation. The definitive analysis of the radiation resistance of MaS-3 was beyond the scope of this paper and could be subject for further research. The cell line showed a very good response to platinum-based chemotherapy, Gemcitabine, and 5-Fluorouracil in the applied concentrations. Though Cisplatin is clearly superior to the use of Oxaliplatin. Additionally, the cell line is responsive to Etoposide, but requires a relatively high dose. These findings are concordant with the case reports by Froehner et al.^22^ and Nomikos et al.^23^. The treatment with Ifosamide as suggested by the case series from Spiess et al.^24^ could not be evaluated by our in vitro experiments as this agent requires hepatic metabolism. Maybe further in vivo experiments could re-evaluate this treatment option. The same authors also reported a case in the series where the carcinosarcoma was treated with methotrexate. MaS-3 had a low IC50 of Methotrexate, but the drug effect did not increase within the therapeutic range, while other substances seem to be more effective^24^. Maybe this was also due to the selected subpopulation through neoadjuvant therapy or a different set of mutations. However further studies will be required to elucidate the chemoresistance mechanisms in patients with this high-grade disease.

Our proteomic analysis aligned the MaS-3 cells more closely with actual carcinosarcoma than with corresponding non-malignant tissue. This outcome further establishes MaS-3 as a suitable in vitro model of carcinosarcoma.

As 3D cell culture models are increasingly used, we assessed the ability of MaS-3 to form spheroids in vitro. As described, the cell line is able to from 3D structures with a distinct border. Although the volume of the spheroids was decreasing over time and some cells detached on the edges, a clear boundary with live cells was always observable during the investigated period. Hence we conclude that MaS-3 is suitable for 3D experiments with the examined method. The optimal seeding density seems to be around 3000–6000 cells.

We successfully established the first cell primary cell line of a bladder carcinosarcoma. MaS-3 resembles the aggressive nature of the original tumour and showed a good response to various treatment regimes. Furthermore, the present study gives relevant information on the pathophysiology of this cell line especially on the genomic, mutational and protein level. In addition, we proved the suitability of the cell line for 3D in vitro experiments. Hopefully, this cell line will help other researches to further elucidate and understand the carcinogenesis and treatment of this rare cancer.

## Materials and methods

### Patient characteristics

The study commenced after review and approval by the Institutional Review Board of the Medical Faculty Mannheim of the University of Heidelberg (Ethical application no: 2012-293 N-MA) and in accordance with all relevant guidelines and regulations. The patient gave informed and written consent before surgical removal of his tumour. The cell line was established from a 86 year-old caucasian male who initially presented with gross haematuria and urinary tract infection. A CT-scan revealed a pelvine tumour transgressing the bladder without infiltrating adjacent organs. In the subsequent CT-guided biopsy, the tumour was pathologically diagnosed as a carcinosarcoma with most probably originating from the bladder (GATA-3 positivity).

After presentation of the case in our interdisciplinary tumour board a neoadjuvant radiotherapy was recommended to downsize the tumour and was performed the following month with a single dose of 1.8 Gy and a total dose of 50.4 Gy to the tumour and the surrounding tissues. The tumour showed a good response and hence a radical pelvic tumour resection with lymphadenectomy was indicated. The resection specimen was histopathologically staged ypT3b; L1 V1 Pn1. No complications occurred after operation. However, the patient deceased 5 months after the procedure due to recurrence of the tumour.

### Cell culture condition

The tissue sample was directly obtained from the operation theatre immediately after the resection of the tumour and finely minced using scalpels into cubes of approximately 1 mm^3^. Neither enzymatic nor mechanical dissociation of the tumour cells was performed as described in Ref.^25^. The tissue pieces were cultured with RPMI medium (Sigma-Aldrich, United Kingdom) supplemented with 1% penicillin–streptomycin-solution (10,000 U/ml penicillin and 10 mg/ml Streptomycin; Sigma-Aldrich, United Kingdom) and initially 20% FCS (Gibco, Argentina) in the outgrow phase. After the third passage, the amount of FCS was reduced to 10%. The cells were maintained at 37 °C in a humidified atmosphere at 5% CO_2_. Medium was replaced every 2 days. The cell line was routinely tested for mycoplasma before experiments . All experiments for this study were performed between the fourth and thirteenth passage. As this sample was the third in our sarcoma study, we named the cell line MaS-3 (**Ma**nnheim **S**arcoma).

### Cell doubling time

Doubling time was assessed by counting the number of viable cells derived from freshly trypsinized monolayers using the Luna automated cell counter system (Logos Biosystem, South Korea) in accordance to the manufacturer’s protocol. Debris smaller than 6 µm was excluded from the measurements. Cell viability was determined by trypan blue staining. 25,000 Cells were seeded in duplicates in 6- well plates with 2 ml medium per well and counted after 12, 24, and 48 h. Each well of the plate was counted three times. Cell culture media was changed every 24 h. The doubling time was calculated from the logarithmic growth curve using the following formula: v = lgN − lgN0/lg2 (t − t0), with doubling time = 1/v, where N = number of cells and t = time.

### Morphology and immunocytochemistry

Growth pattern and cell morphology was determined in vitro using a Zeiss phase-contrast microscope AxiovertA1 (Zeiss, Jena, Germany). For immunohistochemical assays were performed on formalin fixed paraffin-embedded MaS-3 cells. Briefly, freshly trypsinized MaS-3 cells from a T75 flask were 3 times washed with DPBS (Gibco) and centrifuged at 300 g to a pellet. For cytoblock preparation, this pellet was fixed in formalin incorporated into agarose, and subsequently paraffin-embedded. For immunohistochemical staining, the following antibodies and concentrations were used: pan-cytokeratin (1:1000; clone AE1/AE3, cat # M3515, Dako), vimentin (1:400; clone SP20, cat # RM-9120-s, Thermo Fisher Scientific), actin (1:200; clone 1A4, cat # M0851, Dako), LCA/CD45 (1:700; clone 2B11 PD7/26, cat # M0701, Dako), GATA3 (1:100; clone L50 823, cat # 390 M 16, Medac). Detection was done using the EnVision Detection System, Peroxidase/DAB, Rabbit/Mouse (cat # K5007, Dako). All stainings were validated by internal and/or external positive controls as well as negative control specimens.

### Cytogenetics and multicolour fluorescence in situ hybridization analysis

Cytogenetic analyses of 20 Giemsa (G)-banded Metaphases were interpreted according to the International System for Human Cytogenetic Nomenclature (ISCN 2016)^26^. G-banding analysis was combined with multicolour-FISH (m-FISH) analysis according to the manufacturer’s instructions (Metasystems, Germany)^27^.

The composite karyotype (cp) contains all clonally occurring/observed abnormalities and gives a range of chromosome number in the metaphases. The total number of cells in which the clonal changes were observed is given in square brackets after the karyotype, preceded by the symbol cp. Each of the abnormalities has been analysed in at least two metaphases, but there is no cell with all abnormalities (ISCN 2016). Sporadic chromosomal aberrations (observed only in one metaphase) are not mentioned.

### DNA fingerprinting (STR Typing of genomic DNA)

STR DNA profiling was carried out using fluorescent PCR in combination with capillary electrophoresis as described previously^28^. Using different alternate colours, the PowerPlex VR 1.2 system (Promega, Mannheim, Germany) was modified in order to run a two-color DNA profiling allowing the simultaneous single-tube amplification of eight polymorphic STR loci and Amelogenin for gender determination. STR loci of CSF1PO, TPOX, TH01, vWA and Amelogenin were amplified by primers labelled with the Beckman/Coulter dye D3 (green; Sigma-Aldrich, Germany), while the STR loci D16S539, D7S820, D13S317 and D5S818 were amplified using primers labelled with D2 (black). All the loci except the Amelogenin gene in this set are true tetranucleotide repeats. All primers are identical to the PowerPlexVR 1.2 system except the fluorescent colour. Data were analysed with the CEQ 8800 software (Beckman-Coulter, Germany), which enables an automatic assignment of genotypes and automatic export of resulting numeric allele codes into the reference DNA database of the DSMZ.

### Chemosensitivity

For viability evaluation after chemotherapeutic treatment 5000 cells were seeded into 96-well plates in sextuplicates. One day post seeding cells were treated with following chemotherapeutics 5-Fluorouracil, Etoposide, Gemcitabine, Oxaliplatin, Irinotecan and Methotrexate. Clinically widely used Ifosfamide was also evaluated but it proved to be unsuitable for in vitro experiments as it needs to be metabolized within the liver to produce the active metabolites. Untreated MaS-3 cells were used as a negative control. Viability of the cells was assed 48 and 72 h post treatment via MTT assay. Briefly, 20 µl of 5 mg/ml 3-(4,5-dimethylthiazol-2-yl)-2,5-diphenyltetrazoliumbromide (MTT) solution was added to each well and incubated for 4 h at 37 °C. The MTT formazan was dissolved in DMSO and absorbance was measured at 560 nm with reference at 670 nm and expressed as relative values compared to untreated control cells (Tecan infinite M200, Tecan Group).

### Proteomics

Comparative, label-free quantitative proteome analysis was performed with MaS-3 cells (cultured as reported), parental carcinosarcoma tissue, and the corresponding non-malignant tissue specimen obtained from the surrounding of the carcinosarcoma. Samples were lysed in 100 mM HEPES, 5 mM DTT, 0.1% RapiGest using a Precellys homogenizer, followed by heat incubation (85 °C, 90 min), trypsination, alkylation, acidic RapiGest degradation, and desalting using StageTips as reported in Ref.^29^. Mass spectrometry and data analysis using MaxQuant were performed as described in Ref.^29^.

### 3D cell culture

For 3D cell culture 1000, 2000, 3000, 4000, 8000 and 16,000 cells were seeded in quadruplets in 20% methylcellulose (Sigma-Aldrich, United States) enriched media in a 96-well U-bottom plate and centrifuged at 4000 g for 5 min. The diameter of the resulting spheroids was assessed in 2 dimensions every 24 h under a phase contrast microscope. The volume of the spheroids was calculated using the following formula:

$${\text{V}} = 2/3*\uppi *{\text{r}}^{3} ,\quad {\text{where}}\;{\text{r}} = 1/2\surd ({\text{d}}_{{\text{x}}} *{\text{d}}_{{\text{y}}} ).$$

After 96 h media was changed and 1:250 Calcein AM (1 mM, PromoKine) and 1:10 Propidium iodide (1 mg/ml, Sigma-Aldrich) staining was added. Cells were then observed under fluorescence (DM IRB, Leica).

### Statistical analysis

For statistical analysis we used Excel 2016 (Microsoft), JMP 13 (SAS Institute) and Graph Pad Prism version 6.01. Statistical significance was defined as a *p* value < 0.05 in in single factor ANOVA.

## Supplementary information

Supplementary information.
