# Supplementary material for: First report on establishment and characterization of a carcinosarcoma tumour cell line model of the bladder
Source: Sci Rep. 2021 Mar 16;11:6030. doi: 10.1038/s41598-021-85400-5 (PMC7971026; doi:10.1038/s41598-021-85400-5)
Supplement: Supplementary file 1 — Supplementary information. [file 41598_2021_85400_MOESM1_ESM.pdf]

First report on establishment and characterization of a carcinosarcoma tumour cell line model of the bladder

Johannes Eberhard[1]	(johannes.eberhard@umm.de),
Daniela Hirsch[2]	(daniela.hirsch@umm.de),
Oliver Schilling[3,]	(oliver.schilling@mol-med.uni-freiburg.de),
Wilhelm Gerhard Dirks[4]	(wdi@dsmz.de),
Feng Guo[1]	(Feng.Guo@medma.uni-heidelberg.de),
Alice Fabarius[5]	(alice.fabarius@medma.uni-heidelberg.de),
Felix Rückert[1*]	(felix.rueckert@umm.de),
Christoph Reissfelder[1]	(christoph.reissfelder@umm.de),
Peter Hohenberger[6]	(peter.hohenberger@umm.de),
Prama Pallavi[1]	(Prama.Pallavi@medma.uni-heidelberg.de)

[1] Surgical Department, University Hospital Mannheim, Heidelberg University, Mannheim, Germany

[2] Institute of Pathology, University Hospital Mannheim, Heidelberg University, Mannheim, Germany

[3] Institute of Surgical Pathology, University Medical Center Freiburg, Faculty of Medicine - University of Freiburg, Freiburg, Germany

[4] Department of Human and Animal Cell Lines, Leibniz-Institute DSMZ-German Collection of Microorganisms and Cell Cultures, Braunschweig, Germany

[5] Department of Haematology and Oncology, University Hospital Mannheim, Heidelberg University, Mannheim, Germany

[6]Division of Surgical Oncology and Thoracic Surgery, University Hospital Mannheim , Medical Faculty Mannheim, University of Heidelberg, Germany

Corresponding Author:
Felix Rückert
Surgical Department
University Hospital Mannheim
Theodor-Kutzer-Ufer 1-3
68167 Mannheim, Germany
Tel: +49 621 383 2267
Email: felix.rueckert@umm.de

Supplementary Information

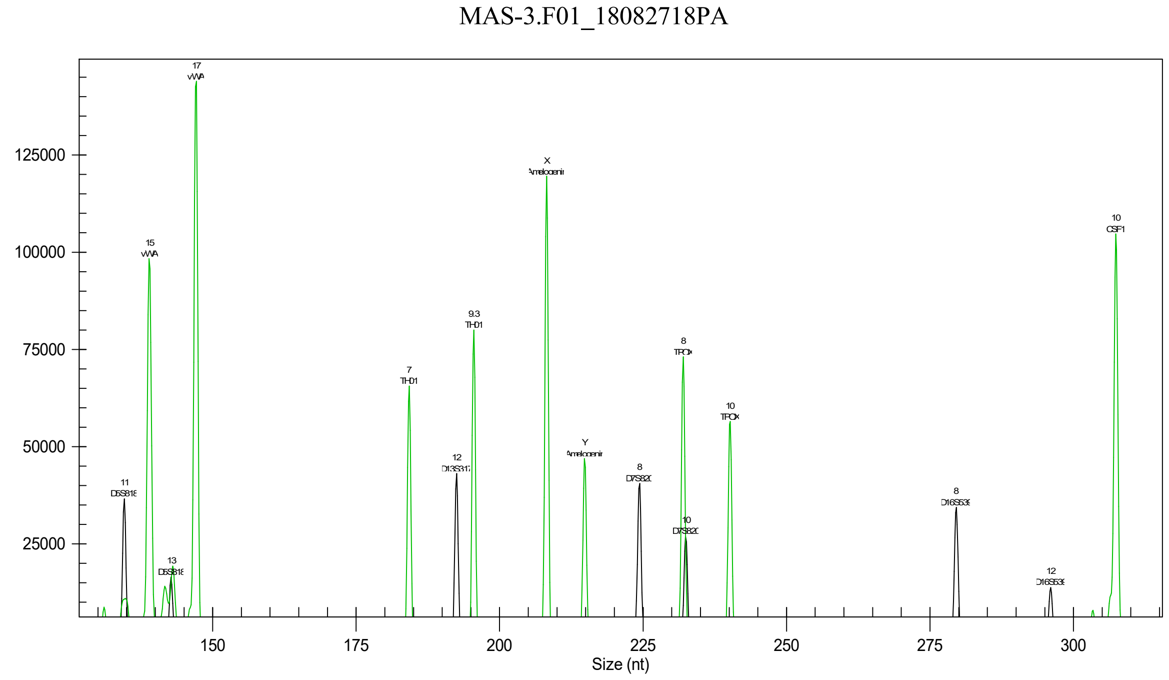

Supplementary Figure 1: Results of ID panel (CODIS/ESS) for MaS-3 and original tumour tissue. Electropherogram of cell line MaS-3. An electropherogram of nonaplex STR profilewas generated of genomic DNA of the cell line MaS-3 is shown. Numbers written above the STR loci indicate the respective alleles. A comprehensive search within the international STR reference database of DSMZ revealed uniqueness of the STR profile of MaS-3.

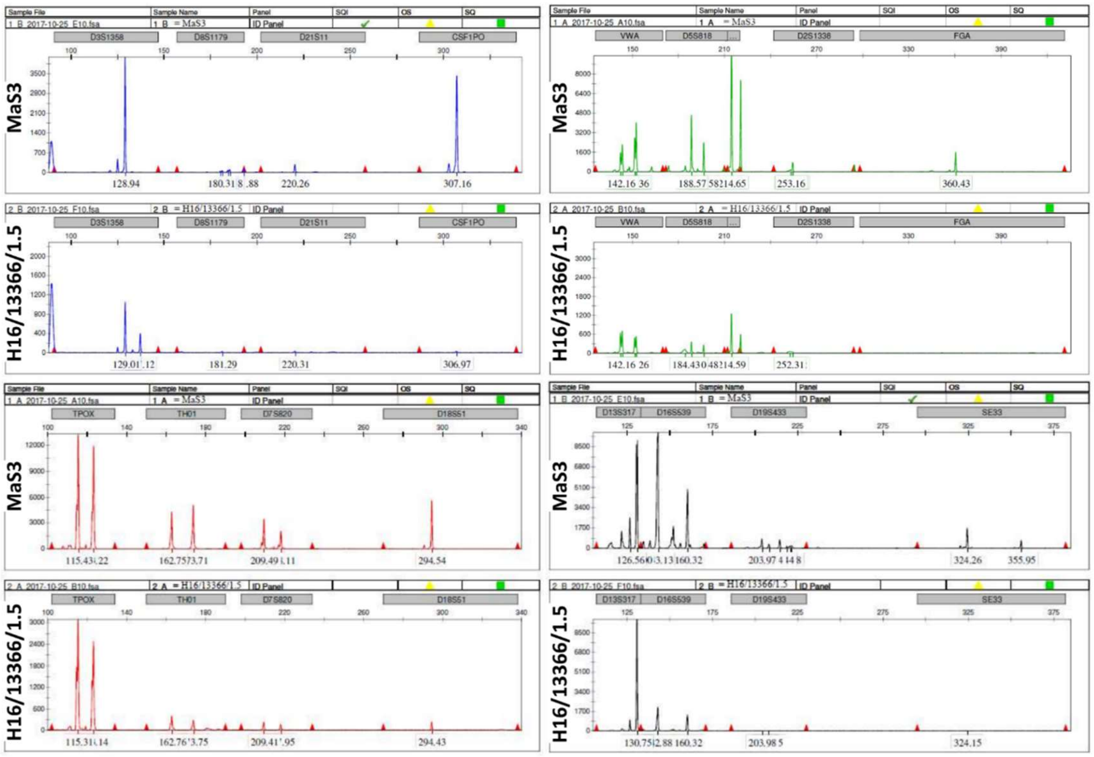

Supplementary Figure 2: ID Panel H16/13366/1.5 (carcinosarcoma, original tumour tissue) and newly established cell line MaS-3. STR profile of the original tumour and derived cell line MaS-3 match sufficiently indicating MaS-3 originates from the patient.

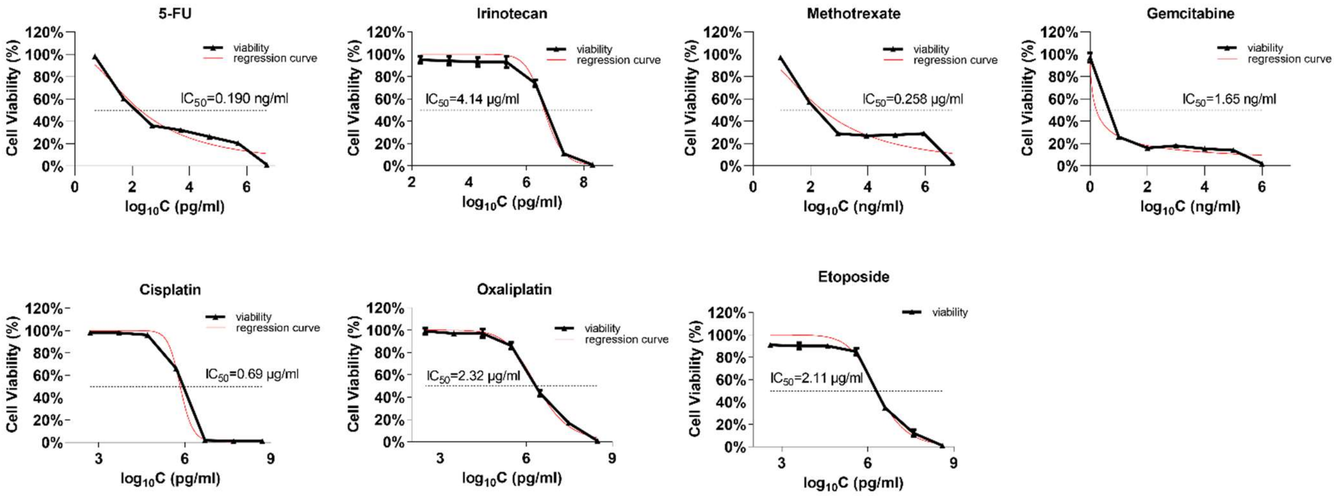

Supplementary Figure 3: IC50 dose response curves of MAS-3 to 72h treatment 5-Flourouracil, Irinotecan, Methotrexate Gemcitabine, Cisplatin, Oxaliplatin and Etoposide as calculated by non-linear regression in GraphPad Prism

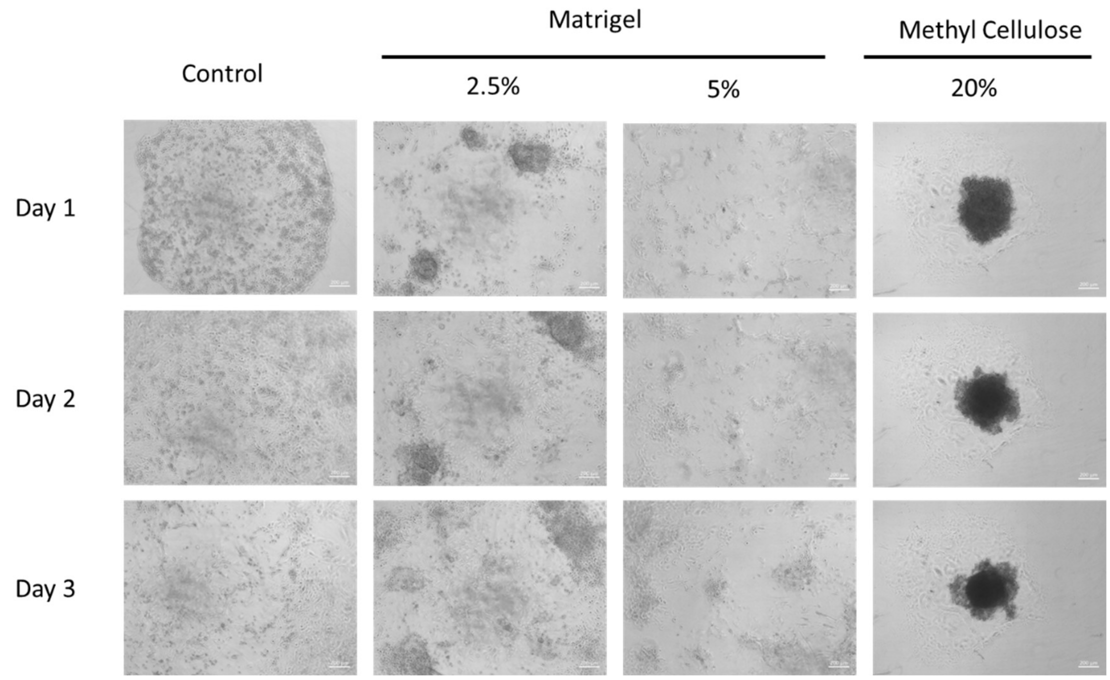

Supplementary Figure 4: Spheroid formation. 4000 MaS-3 cells were seeded into an ultra low attachment 96 well plate with medium suplemented with matrigel (2.5%, 5%) or methyl cellulose (20%) or without any supplements. Spheroids were obtained by centrifugation at 2000g.Pictures were obatined with Zeiss Axiovert imaging System (5 × objective) at day 1-day 3 after seeding.
